# Barriers in the brain: resolving dendritic spine morphology and compartmentalization

**DOI:** 10.3389/fnana.2014.00142

**Published:** 2014-12-04

**Authors:** Max Adrian, Remy Kusters, Corette J. Wierenga, Cornelis Storm, Casper C. Hoogenraad, Lukas C. Kapitein

**Affiliations:** ^1^Cell Biology, Department of Biology, Faculty of Science, Utrecht UniversityUtrecht, Netherlands; ^2^Department of Applied Physics, Eindhoven University of TechnologyEindhoven, Netherlands; ^3^Institute for Complex Molecular Systems, Eindhoven University of TechnologyEindhoven, Netherlands

**Keywords:** dendritic spine, super-resolution microscopy, compartment, diffusion, modeling

## Abstract

Dendritic spines are micron-sized protrusions that harbor the majority of excitatory synapses in the central nervous system. The head of the spine is connected to the dendritic shaft by a 50–400 nm thin membrane tube, called the spine neck, which has been hypothesized to confine biochemical and electric signals within the spine compartment. Such compartmentalization could minimize interspinal crosstalk and thereby support spine-specific synapse plasticity. However, to what extent compartmentalization is governed by spine morphology, and in particular the diameter of the spine neck, has remained unresolved. Here, we review recent advances in tool development – both experimental and theoretical – that facilitate studying the role of the spine neck in compartmentalization. Special emphasis is given to recent advances in microscopy methods and quantitative modeling applications as we discuss compartmentalization of biochemical signals, membrane receptors and electrical signals in spines. Multidisciplinary approaches should help to answer how dendritic spine architecture affects the cellular and molecular processes required for synapse maintenance and modulation.

## Introduction: dendritic spines

The dendritic compartment of a neuron receives input from thousands of upstream neurons via synapses. The majority of excitatory inputs in the central nervous system are located at dendritic spines. Spines are micron-sized protrusions along the dendritic shaft and have first been described about a century ago by Ramón y Cajal (1888). They are composed of a spine head and a thin spine neck that connects them to the dendritic shaft. Typical dimensions are ~<1 μm for the head diameter, and a ~100 nm wide and ~1 μm long spine neck, but notable differences in spine morphology exist (Bourne and Harris, 2008). Based on electron microscopy (EM), three shape categories have been defined: thin, filopodia-like protrusions (“thin spines”), short spines without a well-defined spine neck (“stubby spines”) and spines with a large bulbous head (“mushroom spines”) (Bourne and Harris, 2008). Importantly, spine shape is not static, but can change, even throughout adulthood, reflecting the plastic nature of synaptic connections. For example, neuronal activity *in vitro* and experience *in vivo* can alter spine morphology (Holtmaat and Svoboda, 2009). Changes in spine size are thought to be generally correlated with changes in the strength of the excitatory synapse (Schikorski and Stevens, 1999; Arellano et al., 2007). Such functional and structural changes of spines and synapses are believed to be at the core of learning and memory in the brain (Yuste and Bonhoeffer, 2001; Holtmaat and Svoboda, 2009; Kasai et al., 2010).

The actin cytoskeleton plays a key role in shaping dendritic spines and is critically important for numerous processes that contribute to the plasticity of synaptic function (Matus, 2000; Hering and Sheng, 2001; Luo, 2002; Ethell and Pasquale, 2005; Hotulainen and Hoogenraad, 2010). The rapid polymerization and depolymerization of actin filaments produces protrusive forces that can quickly change neuronal morphology (Kessels et al., 2011). For example, during spine enlargement, rapid actin polymerization provides the mechanical force required for pushing out the spine membrane (Bosch and Hayashi, 2012). In addition, the actin cytoskeleton provides tracks for myosin-based transport of various cellular materials in and out of spines, including AMPA-type glutamate receptors (Kneussel and Wagner, 2013).

The mechanisms through which spine shape affects its function are not yet fully understood. At its minimum, morphological changes associated with synaptic modulation could just be a secondary effect of altered actin dynamics required to more directly modulate synapse functioning or actin-based transport. Nevertheless, modeling studies have often emphasized the interesting effects that shape can have on the diffusion of proteins, calcium ions and other signaling molecules (Holcman and Schuss, 2011). A small neck should slow diffusion and result in functional compartmentalization by preventing signaling molecules to escape from the spine. In addition, more recent modeling studies report that shape should also affect lateral diffusion of proteins embedded in the plasma membrane (Kusters et al., 2013). Several studies have indeed reported evidence for compartmentalization, but the extent to which this was governed by shape alone could often not be directly assessed because the limited resolution of live-cell light microscopy did not allow to directly correlate diffusion dynamics and spine shape. Recent breakthroughs in fluorescence microscopy allow imaging at resolutions below the diffraction limit, allowing to directly explore how spine shape affects diffusion of cytoplasmic or membrane-embedded molecules (Takasaki and Sabatini, 2014; Tønnesen et al., 2014). In this review, we first discuss existing and emerging technologies to image spine morphology. We then present existing evidence for the compartmentalization in spines. Finally, we discuss how different aspects of spine shape contribute to compartmentalization, with an emphasis on recent modeling studies exploring the influence of shape on lateral diffusion in the membrane.

## Imaging spine morphology

Ramón y Cajal (1899-1904) discovered dendritic spines using light microscopy of neurons stained using Golgi impregnation and he suggested these small protrusions to be sites of neuronal signal transmission. His hypothesis was confirmed with the development of EM during the interwar, which allowed imaging at much higher resolution (Gray, 1959). Subsequent refinements of this technology, especially the careful analysis of series of thin tissue sections in serial-sectioning EM, allowed a full morphological description of dendritic spines and have provided many beautiful insights into spine architecture (Bourne and Harris, 2008). Serial-sectioning EM directly visualizes all tissue surrounding spines as well as the structure of the postsynaptic specialization and has been used to identify precise morphological changes upon specific stimuli (Bourne and Harris, 2008). However, the use of EM also has several limitations. First of all, sample preparation procedures and imaging conditions prevent imaging of living tissue. In addition, different preparation procedures can easily introduce artifacts (Bourne and Harris, 2012) and also the labeling of specific proteins has so far remained challenging and very inefficient. Therefore, to study dynamics of spines or specific proteins associated with spines, live-cell fluorescence microscopy is the method of choice.

### Conventional live cell imaging

Both laser-scanning and spinning disk confocal microscopy are standard techniques to study spine dynamics in dissociated neurons. For imaging in tissue, however, these techniques impose several limitations. Visible light penetrates poorly into tissue and is quickly distorted, resulting in a rapid loss of resolution with increased focus depth because the focus size is no longer diffraction-limited. In addition, many focal planes need to be imaged sequentially to reconstruct complete neurons in three dimensions. Because exposure to excitation light is not restricted to the plane in focus, this results in increased phototoxicity and photobleaching, limiting sample life time and signal intensity.

Two-photon microscopy overcomes both of these limitations through the use of a pulsed infrared light source that excites fluorophores by the combined energy of two photons arriving on the sample nearly simultaneously (Denk et al., 1990; Svoboda et al., 1996). Infrared light is much less distorted and penetrates deeper into the sample compared to visible light. As two-photon excitation efficiency scales quadratically with excitation intensity, it is largely limited to the focus plane and prevents photobleaching of out-of-focus planes. Two-photon microscopy rapidly became the method of choice for deep tissue imaging and has even enabled intravital brain imaging in mice (Svoboda and Yasuda, 2006). However, despite its unique advantages, the resolution of two-photon microscopy is still inherently limited by diffraction to 400–500 nm. Therefore, several studies have combined two-photon live imaging with *post hoc* serial sectioning EM to examine the microstructure of spine, for example to directly demonstrate synapse formation associated with the emergence of a new spine during live imaging (Trachtenberg et al., 2002; Holtmaat et al., 2006; Bourne and Harris, 2012). As this requires fixing the sample, this approach might not detect all morphological changes that occur upon specific stimuli and is prone to morphological artifacts. To better study these processes, live-cell imaging beyond the diffraction limit is required.

### Live-cell imaging beyond the diffraction barrier

The diffraction of light limits the ability of microscopes to resolve the location of two objects that are located closer to each other than approximately half the wavelength of the light used for imaging. For conventional fluorescence microscopy using visible light this limit lays around 200–300 nanometers. Over the last years, different technologies have allowed fluorescence microscopy at a resolution below the diffraction limit (Hell, 2007). Dendritic spines have frequently been used for proof-of-principle applications of these techniques, because of their small size and physiological relevance. Indeed, careful analysis of spine morphologies using superresolution microscopy has demonstrated that conventional light microscopy methods overestimate the amount of stubby spines in acute and organotypical slice cultures (Tønnesen et al., 2014). Here, we highlight several techniques that have recently contributed to novel insights into spine morphodynamics and synapse architecture.

Stimulated Emission Depletion (STED) microscopy was developed as an extension of confocal microscopy. The conventional excitation beam is complemented with a depletion beam that forms a donut shaped spot surrounding the focus of the excitation beam (Klar et al., 2000; Hell, 2007). The wavelength of the depletion beam is chosen within the tail of the emission spectrum of the imaged fluorophore. It brings fluorophores excited by the excitation laser back to their ground-state by inducing stimulated emission at exactly the wavelength of the depletion beam. As a result, fluorescence emission at all other wavelengths of the emission spectrum is restricted to the center of the donut. Importantly, the size of this zone is not limited by diffraction. Therefore, scanning the lasers with very small steps over the sample improves the resolution of the final image up to 50 nm (Klar et al., 2000; Hell, 2007).

The first STED images of spines in organotypical slices expressing yellow fluorescent protein (YFP) in a sparse subset of neurons were published in 2008 (Nägerl et al., 2008). Neck diameters of spines located at 0–10 μm depth were originally measured to be on average ~40% reduced compared to confocal imaging (Nägerl et al., 2008), whereas more recent measurements have found neck diameters as low as 51 nm in organotypic cultures and 59 nm in acute slices (Tønnesen et al., 2014). Recently, STED microscopy has also been established *in vivo* in mouse brain (Berning et al., 2012).

The STED principle can also be applied to two-photon microscopy (Moneron and Hell, 2009). However, the depletion wavelength needs to be within the (visible) emission spectrum and is therefore prone to distortions. Nevertheless, it has been successfully applied to dendritic spines in organotypical slices, resulting in 60–150 nm lateral resolution at 50–100 μm depths, but without improving the axial resolution (Ding et al., 2009; Bethge et al., 2013; Takasaki et al., 2013). In addition, two-color detection has been established using spectral unmixing of either pairs of organic dyes (Tønnesen et al., 2011) or the fluorescent proteins YFP and GFP (Bethge et al., 2013). None of the techniques described here improve the axial resolution. However, development of three-dimensional depletion patterns and compensation of optical distortions through adaptive optics promise improvements in the near future (Gould et al., 2012; Loew and Hell, 2013).

Inducing stimulated emission requires very high light intensities, which can induce artifacts and phototoxicity in the imaged sample. To circumvent this, a comparable technique reduces the size of the confocal volume using a specifically engineered fluorescent protein that can transition to a non-fluorescent dark state (RESOLFT: reversible saturable/switchable optical transitions; Grotjohann et al., 2011). This approach requires orders of magnitude of less light intensity and has been demonstrated on living brain slices (Testa et al., 2012). Novel probes are currently being developed that should allow two-color RESOLFT of dendritic spines (Lavoie-Cardinal et al., 2014; Shcherbakova et al., 2014).

Another set of powerful techniques to achieve resolutions beyond the diffraction barrier uses switchable fluorophores or special imaging conditions to ensure that only a small, random subset of fluorophores in the sample is emitting at any given time (Huang et al., 2009). Because these fluorophores are then distributed sparsely enough to be clearly separated, their positions can be obtained from their point spread function with 1–10 nm accuracy. Repeating this procedure thousands of times for different subsets of fluorophores in the region of interest eventually allows reconstructing a superresolved image from the calculated positions. This basic concept of repetitive detection of small subsets has been applied in many different ways and these techniques are collectively referred to as single-molecule localization microscopy (SMLM), of which the most prominent variants are known as PALM, STORM and dSTORM (Huang, 2010). These techniques are often used on fixed samples, because the temporal resolution is limited by the repetitive detection and the required excitation intensities are high. Nevertheless, several groups have succeeded in live-imaging of dendritic spines using these techniques: spine morphology has been probed using labeled antibodies against membrane-bound proteins (Giannone et al., 2010; Ries et al., 2012), using genetically encoded fluorophores that either directly label or transiently bind to actin (Frost et al., 2010; Izeddin et al., 2011), or using a lipophilic cyanine dye that labels the plasma membrane (Shim et al., 2012). In all cases, live super-resolution microscopy requires some thoughtful compromises between temporal and spatial resolution (Frost et al., 2012).

## Dendritic spines form dynamic compartments

In principle, there may be several advantages of having substructures like dendritic spines containing synapses along the dendrite. First of all, spines might facilitate connectivity by bridging the physical gap between slightly distant axons and dendrites. However, not all neurons have spines (e.g., stellate neurons) and they can receive excitatory input directly on their shafts (Anderson et al., 1994). It is therefore likely that spines have additional functions. Ever since their discovery by Ramón y Cajal (1899-1904), it has been suggested that spines may play a role in the compartmentalization of synaptic signals. Such compartmentalization may facilitate spine-specific plasticity and thereby regulate the individual strength of synaptic connections (Yuste and Denk, 1995; Matsuzaki et al., 2004; Holtmaat and Svoboda, 2009; Araya et al., 2014). Compartmentalization of spines has been reported on three levels that we discuss in detail in this section: (1) from a molecular and cell biological perspective, signaling cascades elicited by synaptic stimulation may be confined to single spines, making them biochemical signaling compartments that confine structural plasticity to individual spines; (2) spines may also compartmentalize neurotransmitter receptors, both by opposing their diffusion out of spines and by maintaining spine-selective intracellular storage pools, in order to directly regulate the sensitivity of a synapse to stimulations; and (3) lastly, spines may serve as an electrical compartment, playing a role in the processing of synaptic depolarization from synapses along the dendrite.

### Compartmentalization of biochemical signaling

The compartmentalization of spines is most easily studied by measuring the extent to which fluorescent dyes, specific proteins or ions exchange between a spine and the parent dendrite. We first discuss studies that examined the cytoplasmic coupling between spines and the dendritic shaft as a general measure of spine head isolation. In addition, we review the evidence for specific biochemical compartmentalization of calcium ions and signaling molecules.

#### Diffusional coupling between spine and dendrite

Using two-photon fluorescence recovery after photobleaching (FRAP) microscopy on hippocampal neurons filled with fluorescent dyes in cultured slices, early studies found that spines can indeed compartmentalize cytoplasm as fluorescence recovery rates in dendritic spines are significantly lower than in the shaft (Svoboda et al., 1996). Repeated activation of a photo-activatable variant of GFP (PA-GFP) in individual spines showed substantial variation in the cytoplasmic coupling of individual spines over time that may be regulated by neuronal activity. In a small population of spines, no exchange of soluble fluorescent proteins between shaft and spine heads could be measured during a period of several minutes (Bloodgood and Sabatini, 2005). Whereas in these earlier studies the exact relation between spine shape and cytoplasmic coupling could not be resolved, recent experiments have used STED microscopy to correlate spine morphology and cytoplasmic diffusion kinetics (see Section Spine Necks as Barriers).

In addition to the exchange of soluble dyes and fluorescent proteins between spines and dendrites, the diffusion of calcium ions has also been studied extensively (Bloodgood and Sabatini, 2007a). Calcium ions play a crucial role in initiating downstream signaling during long-term potentiation (LTP) and depression (LTD) and influx of calcium is both necessary and sufficient for structural synaptic plasticity (Pettit et al., 1994; Lledo et al., 1995; Otmakhov et al., 2004). Pioneering two-photon microscopy of calcium dynamics in slices of hippocampal neurons revealed that synaptic stimulation results in accumulation of calcium ions in single spines (Yuste and Denk, 1995). The extent to which the diffusion kinetics of calcium ions are regulated by spine morphology and neck width in particular is debated in the literature. Importantly, when calcium is bound to buffering proteins like calmodulin, the diffusion of the resulting complex is more sensitive to spatial constraints than single ions because of its larger size (Sabatini et al., 2002; Tønnesen et al., 2014). In addition to diffusion into the dendritic shaft, calcium can also be removed from spines by absorption into the smooth endoplasmic reticulum located in spines or by Na/Ca exchangers located in the plasma membrane (Sabatini et al., 2002). These processes help to confine transient calcium ions to the spine head. Nevertheless, morphology does play a role, as long and thin necks prevent the diffusion of calcium, whereas shorter and thicker necks allow for better diffusional coupling with the dendrite (Majewska et al., 2000; Holthoff et al., 2002; Sabatini et al., 2002; Korkotian et al., 2004; Noguchi et al., 2005).

Other factors influencing the local calcium concentrations in spines are the surface to volume ratio of spines and the localization of calcium-permeable ion channels (Sabatini et al., 2002). If these ion channels were distributed equally throughout the plasma membrane, one would expect a higher effective concentration of these channels in spines compared to dendrites as the surface-to-volume ratio of the former is higher. This should theoretically lead to a higher influx of calcium in spines than in the surrounding dendrite (Sabatini et al., 2002). Such an effect is strengthened by the existence of classes of voltage gated calcium channels that exclusively localize to spines but not dendrites and cooperate with other calcium channels to shape local depolarization and synaptic plasticity (Bloodgood and Sabatini, 2007b; Bloodgood et al., 2009).

#### Spatial restriction of signaling domains

Calcium ions in dendritic spines have an important function in activating signaling cascades that underlie and regulate synaptic plasticity (Kennedy et al., 2005). Their retention in an individual stimulated spine may thus be important to induce downstream signaling and structural plasticity in a synapse-specific manner. CaMKII is a calcium-activated kinase involved in structural plasticity by remodeling of the postsynaptic density (Yoshimura et al., 2002), rearrangement of the actin cytoskeleton (Okamoto et al., 2007) and maintenance of spine enlargement (Matsuzaki et al., 2004; Lee et al., 2009). Downstream of CaMKII, Ras and Rho GTPases are important for regulating spine morphology (Ramakers, 2002; Saneyoshi et al., 2010) and synaptic strength (Zhu et al., 2002; Patterson et al., 2010). It is thought that Rho activation causes spine loss and shrinkage by inhibiting actin polymerization, whereas Cdc42 and Rac activation increase the number of spines by promoting actin polymerization. The precise crosstalk and integration is however not completely understood (Kennedy et al., 2005).

**Figure 1:**
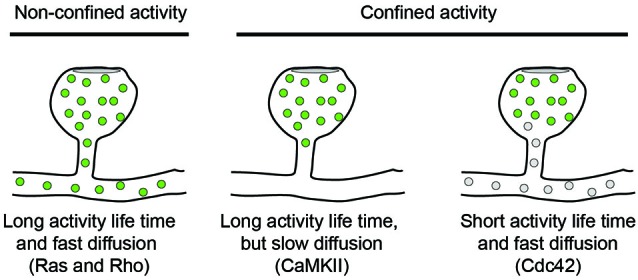
**Diffusion models for signaling molecules in spines**. The spread of active signaling molecules (green) with long activity life-times depends on their diffusion rate. Limiting the activity life time of signaling molecules is an orthogonal approach to confine signaling activity to individual spines.

To explore the activity of signaling molecules, activity sensors can be used in which the amount of Förster Resonance Energy Transfer (FRET) between two fluorophores is different between active and inactive conformations. FRET is the process in which an excited donor molecule transfers energy to an acceptor fluorophore, whose excitation spectrum overlaps with the emission spectrum of the donor. The efficiency of this energy transfer is very sensitive to the distance between both fluorophores, which should be within the 2–5 nm range. Energy transfer can be detected either by the appearance of red-shifted emission from the acceptor, or by a decrease in the excited state life time of the donor. In a series of papers, Yasuda and co-workers have used activity reporters for different signaling molecules and measured their fluorescence lifetimes by two-photon microscopy (2P-FRET-FLIM) in cultured hippocampal slices. Additionally, using photoactivatable protein tags, the diffusion kinetics of the same proteins could be measured in spines. This combination of techniques allowed recording activity patterns for CaMKII (Lee et al., 2009), Ras (Harvey et al., 2008), RhoA and Cdc42 (Murakoshi et al., 2011) in dendritic spines following local glutamate uncaging. Intriguingly, these signaling molecules show different activity patterns: while CaMKII and Cdc42 activities are confined to the stimulated dendritic spine (Lee et al., 2009; Murakoshi et al., 2011), Ras and RhoA activities spread along the parent dendrite. Ras activity was shown to invade typically 10–20 neighboring spines in a range of 10 μm along the dendrite, whereas RhoA activity only spread 5 μm and rarely invaded neighboring spines (Harvey et al., 2008; Murakoshi et al., 2011). Thus, despite all being triggered by NMDA-dependent calcium influx, these molecules have quite different signaling ranges. The spread of their signaling activity depends on three factors: (1) the extent and persistence of the upstream activation event; (2) the diffusion rate of the signaling molecule; and (3) its inactivation kinetics, see Figure 1.

Upon single-spine glutamate uncaging, the activation of CaMKII peaked within 6 s and only lasted for a few minutes (Lee et al., 2009). The diffusional coupling of CaMKII with the dendrite was significantly slower, in the range of several minutes (Lee et al., 2009), and additional modeling studies have shown that the effective CaMKII diffusion constant depends heavily on binding to synaptic scaffolds and the actin cytoskeleton in spine necks (Byrne et al., 2011). Thus, because inactivation of CaMKII has faster kinetics than CaMKII diffusion, activity of the kinase is restricted to stimulated spines. It should be noted that these results based on single spine stimulations contrast earlier biochemical studies that reported persistent phosphorylation of CaMKII upon more global induction of LTP (Fukunaga et al., 1995; Barria et al., 1997; Lisman and Zhabotinsky, 2001).

The activation of Ras measured by FRET-FLIM was dependent on CaMKII activation, peaked later, and recovered to baseline level only after 15 min. Its diffusion rate out of the spine was relatively fast, reaching the dendrite within seconds without leaving any immobile fraction in the spine (Harvey et al., 2008). Together, this explains why Ras activity can spread to neighboring synapses.

Both Cdc42 and Rho showed a rapid activity peak 30 s after stimulation followed by decay over 5 min and a sustained lower activity for more than 30 min. Also the diffusion kinetics of both molecules were comparable and similar to Ras diffusion and showed no immobile fraction left in the spine. Nevertheless, Cdc42 activity remained spatially confined, because it had an intrinsic inactivation time constant of 6 s and therefore depends on continuous activation by CaMKII (Murakoshi et al., 2011). In contrast, Rho inactivated five times slower and could therefore spread into the dendrite. Thus, the specific combinations of activity life-time and diffusion kinetics can explain why CaMKII and Cdc42 activities are restricted to spine heads whereas Ras and Rho activities spread along the dendrite.

From the examples of CaMKII, Ras and Rho it becomes clear that the interplay between diffusion and activity of the signaling proteins is highly coordinated in dendritic spines. This is crucial because these signaling events are thought to coordinate local processes in the stimulated spine. CaMKII-Cdc42-Pak signaling increases spine volume and synaptic strength (Murakoshi et al., 2011), while Rho signaling leads to local AMPA receptor integration in the dendrite (Patterson et al., 2010). In addition, Rho-Rock and Ras-ERK signaling pathways lower the threshold for LTP in neighboring spines (Zhu et al., 2002; Harvey and Svoboda, 2007; Harvey et al., 2008; Lee et al., 2009). In potentiated spines, the structural rearrangement of spine morphology during LTP slows the diffusional coupling between spines and dendrites even further (Tønnesen et al., 2014). Taken together, this highly controlled signaling network allows precise spatiotemporal activation and retention of calcium-induced signaling.

### Compartmentalization of membrane-bound receptors

Another aspect of compartmentalization in individual spines is the distribution of membrane and membrane-bound proteins. Controlled addition and removal of AMPA-type glutamate receptors from the postsynaptic density is believed to underlie the changes in synaptic strength during learning and memory formation (Malinow and Malenka, 2002; Sheng and Kim, 2002; Bredt and Nicoll, 2003; Collingridge et al., 2004). Whereas reports based on static EM suggested that glutamate receptors are restricted to synapses (Nusser, 2000), live-cell imaging techniques like FRAP and single molecule tracking changed this view radically (Richards et al., 2004; Triller and Choquet, 2005). Lateral diffusion of receptors through the plasma membrane and activity-triggered exocytosis of receptors from internal endosomal compartments have become generally accepted regulation mechanisms for synaptic plasticity, although their respective contributions have remained unresolved (Passafaro et al., 2001; Borgdorff and Choquet, 2002; Adesnik et al., 2005; Ashby et al., 2006; Park et al., 2006; Ehlers et al., 2007; Heine et al., 2008; Newpher and Ehlers, 2008; Yang et al., 2008; Kennedy et al., 2010; Opazo and Choquet, 2011; Czöndör et al., 2012; Czöndör and Thoumine, 2013). Here, we focus on the contribution of lateral diffusion of glutamate receptors and its regulation during structural plasticity of dendritic spines.

The study of bulk AMPA receptor mobility has been greatly facilitated by the generation of a pH sensitive GFP variant, called superecliptic pHluorin (SEP; Miesenböck et al., 1998; Ashby et al., 2004), whose fluorescence is quenched in the acidified endosomal compartments, but not in the extracellular environment after incorporation into the plasma membrane. Studying the FRAP dynamics of a fusion of SEP with the AMPA receptor subunit GluA2 (SEP-GluA2) revealed that receptor turnover in spines is slower compared to non-spinal plasma membrane and that recovery in the spine neck and base is particularly slow (Ashby et al., 2006). Furthermore, fluorescently-tagged plasma membrane probes in spines of different morphologies showed a faster recovery rate in stubby than in mushroom-shaped spines (Richards et al., 2004; Ashby et al., 2006), indicating that mushroom-shaped spines form a membrane compartment in which diffusion is slowed. Combining SEP-GluA1 photobleaching experiments with glutamate uncaging in organotypic slices revealed that synaptic potentiation of AMPA receptors is achieved by restricting their diffusion out of the synaptic membrane (Makino and Malinow, 2009). In addition, a live SMLM study showed reduced diffusion speeds of plasma membrane markers in spine necks (Shim et al., 2012). Together these data indicate that the spine neck is a general diffusion barrier for membrane-bound proteins which, together with the regulated retention of receptors in the synapse, regulates receptor diffusion in the spine compartment.

Another approach to study receptor dynamics in dendritic spines uses single-particle tracking. Membrane-bound receptor movements are followed with extracellular probes, e.g., antibodies or derived fragments, coupled to fluorescent reporters (Triller and Choquet, 2005). The earliest report used latex beads coupled to GluA2 receptors and found that these receptors reversibly stop at synaptic sites. This is modulated by neuronal activity levels that affect calcium transients in the cell. Calcium elevations were shown to generally slow diffusion and locally accumulate AMPA receptors (Borgdorff and Choquet, 2002). However the large size of the beads (~200 nm) precluded a more detailed analysis of receptor motility in spines and synaptic membrane domains. Fluorescently labelled glutamate receptor antibodies were subsequently used to address diffusion kinetics in synaptic and extrasynaptic regions. A pool of synaptic receptors was shown to be immobile while another synaptic pool and extrasynaptic receptors were rapidly moving. Glutamate stimulation enhances the exchange between these pools and increases overall motility of glutamate receptors (Tardin et al., 2003). Long-term tracking of receptor movements was facilitated by using quantum-dots (QD) as fluorescent probes, which have the advantage of relatively small diameters and good photostability allowing bleaching-free imaging over prolonged times (Dahan et al., 2003). AMPA receptors tagged with QDs were reported to selectively reduce their mobility at active synapse while they freely diffused through non-active synapses (Ehlers et al., 2007). The same study observed reduced exchange of single receptor molecules between spines during stimulation. In addition, recent studies have shown that glutamate receptors localize to submicron scale clusters within the synaptic membrane as shown by various techniques ranging from EM to PALM microscopy (Ehlers et al., 2007; MacGillavry et al., 2013; Nair et al., 2013).

How exactly synaptic recruitment and localization of glutamate receptors are regulated is currently under debate. Whereas previously the cytoplasmic tails of GluA receptors have been shown to differentially regulate receptor diffusion and trapping at synapses (Passafaro et al., 2001), a recent study suggests that truncated receptors void of any cytoplasmic tail can rescue the depletion of endogenous GluA1-3 (Granger et al., 2013). Even though the physiological relevance of these experiments has been debated (Sheng et al., 2013), the observed diffusional trapping of truncated receptors in the spine head is interesting as it requires an intrinsic property of the spine to accumulate transmembrane proteins. Based on results from modeling (Renner et al., 2009) it has been suggested that crowding in the spine head may contribute to this phenomenon (Colgan and Yasuda, 2014). In addition, trapping of receptors may be facilitated by the curvature of plasma membrane in spines (Kusters et al., 2013), as discussed in detail below.

### Compartmentalization of electrical signals

In addition to inducing compartmentalization of biochemical signals and receptors, it has been suggested that dendritic spines may also serve as electrical compartments (Segev and Rall, 1998; Yuste, 2013). As small protrusions connected to a large dendrite, spines may be theoretically described as sealed-end cables with an intrinsic asymmetry in conducting electric signals. This means that voltage signals from the dendrite propagate without attenuation into the spines (Holthoff et al., 2010; Carter et al., 2012; Popovic et al., 2014), but synaptic potentials generated inside the spine head are filtered when they travel to the dendrite (Araya et al., 2006, 2014; Harnett et al., 2012). In addition, the high input resistance of spines may further facilitate synaptic potentials inside spines compared to equally strong synapses onto the dendritic shaft. However, how much of these effects contribute to the compartmentalization of synaptic potentials in spines is strongly debated, as most of the relevant parameters, such as spine neck resistance, are simply not known and experimentally inaccessible at present times.

Because voltage and calcium imaging at single spine resolution has long been technically challenging, the majority of available literature either discusses theoretical work or indirectly calculated spine neck resistances based on diffusional coupling of cytoplasm (Shepherd, 1996; Svoboda et al., 1996; Tsay and Yuste, 2004) and often relies on static morphology data from EM (Harris and Stevens, 1988; Koch and Zador, 1993). The resulting values for the spine neck resistance have varied over a wide range and are strongly influenced by the methods and theoretical models used. Most recently, STED microscopy on dendritic spines in organotypic and acute slices suggested that electric compartmentalization is moderate, but not absent, in most spines (Takasaki and Sabatini, 2014; Tønnesen et al., 2014). For the final answer we will probably need to wait until it is possible to directly measure synaptic potentials in spines and nearby dendrites with voltage-sensitive dyes.

Interestingly, induction of plasticity not only results in an increase in spine size (Matsuzaki et al., 2004; Tanaka et al., 2008), but also in changes in spine shape (Grunditz et al., 2008), with consequences for compartmentalization. It was shown that reduction of spine neck length after synaptic potentiation mediates enhanced electric coupling of spine and dendrite, thereby increasing the influence of the potentiated spine on the dendritic and somatic membrane potential (Araya et al., 2014; Tønnesen et al., 2014). Interestingly, it was suggested that the reduction in electrical compartmentalization occurs while chemical compartmentalization is preserved, reflecting two separate functions of spines within the dendrite.

In addition to passive amplification of synaptic potentials, spines are thought to be able to actively contribute to local membrane voltage. Voltage-dependent ion channels are present within spines and activation of these channels results in a change of local membrane potential. Opening of sodium and calcium channels boosts local depolarization (Araya et al., 2007; Bloodgood et al., 2009; Holbro et al., 2010; Carter et al., 2012; Hao and Oertner, 2012), while opening of potassium channels decreases local input resistance and results in smaller synaptic potentials (Ngo-Anh et al., 2005; Giessel and Sabatini, 2010). These active properties of dendritic spines are thought to play an important role in the interactions between multiple synaptic inputs in dendritic computation (Araya et al., 2007; Harnett et al., 2012).

## Spine morphology as compartmentalization mechanism

We have summarized the evidence for spine-based compartmentalization on three levels: biochemical signaling, membrane-bound receptor dynamics and electrical signaling. All of these levels contribute to proper information processing in the dendritic arbor and are interconnected. However, the exact mechanisms through which spines can regulate different aspects of compartmentalization have remained unclear. Do reduced diffusion rates depend on dedicated barriers imposed by specific protein-based structures, similar to the way in which the axon initial segment forms a barrier for axon entry? Or is the shape of spines sufficient to confine both membrane-based and cytoplasmic diffusion? How exactly do these processes depend on spine neck diameter and spine neck length? In addition, the effect of spine neck constriction on vesicle transport through neck has remained largely unexplored. In this section, we first discuss the role of the spine neck in diffusional coupling with the dendrite and then focus on recent studies showing that spine morphology directly influences lateral diffusion of membrane-bound proteins to and from the synapse. Finally, we discuss the effect of spine shape on vesicular transport into spines.

### Spine necks as barriers

**Figure 2:**
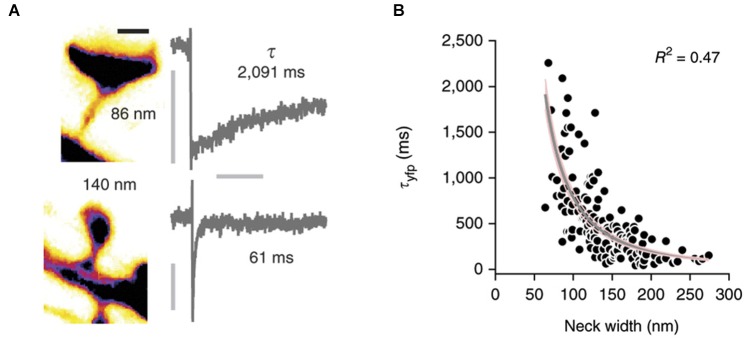
**Correlation of spine morphology and diffusional coupling. (A)** Left: Two dendritic spines filled with soluble fluorophores were imaged with STED microscopy and neck diameters measured with line scans. Scale bar 500 nm. Right: The rate of diffusional coupling (*τ*) of these spines was measured by the recovery of photobleaced fluorophores (FRAP). **(B)** *τ* plotted as function of neck width. Gray line indicates inverse-square fit with 95% confidence interval in pink. Reprinted by permission from Macmillan Publishers Ltd: Nature Neuroscience (Tønnesen et al., 2014), copyright 2014.

Conventional two-photon microscopy has a limited resolution that prevents accurate description of spine shape. Several pioneering studies have recently used STED microscopy (Ding et al., 2009; Bethge et al., 2013; Takasaki et al., 2013) to overcome this problem and studied the correlation between spine morphology and diffusional coupling to dendrites by analyzing the recovery of fluorescence after photobleaching of soluble fluorophores in the spine (Takasaki and Sabatini, 2014; Tønnesen et al., 2014, see Figure 2). Both studies suggest that the recovery time scale *τ* roughly follows what would be expected if diffusion is governed largely by spine geometry:

$$\tau =\frac{V*L}{D*A}$$

where *V* denotes the volume of the spine head, *D* the diffusion coefficient, and *L* and *A* the length and cross-sectional area of the neck, respectively. Indeed, both groups find an inverse relation between spine neck diameter *d* and the recovery time, which appears to follow the predicted inverse quadratic relation (*τ* ∝ 1/*d*^2^). However, whereas the equation suggests a linear increase of the recovery time scale with spine head volume, Takasaki and Sabatini (2014) instead find a weak decrease. Similarly, Tønnesen et al. (2014) report a quadratic dependence on head width *w*, whereas the model predicts a *w*^3^ dependence (assuming a spherical spine head in which *V* ∝ *w*^3^). Interestingly, a fraction of spines strongly deviated from the average trends that were observed (Takasaki and Sabatini, 2014), suggesting that small local constrictions, local protein accumulations and organelle positioning in the neck may create additional diffusion barriers (Arellano et al., 2007; Yuste, 2013). Nonetheless, these important studies demonstrate that the constriction of the spine neck alone has a major impact on crosstalk between spine and dendrite.

Membrane-bound proteins like glutamate receptors are restricted in their passage through the spine neck, as we discussed in the Section Compartmentalization of Membrane-bound Receptors. Such restriction has several causes: in addition to the direct influence of the spine morphology on membrane proteins that we discuss in the next section (Kusters et al., 2013), several cell-biological factors including molecular crowing, corralling and receptor retention in synaptic scaffolds have been studied in recent years. The postsynaptic density is believed to regulate the number of glutamate receptors localized in the synapse and thereby preventing their diffusion out of the spine (Opazo and Choquet, 2011). Additionally, the high density of proteins in the synapse may reduce diffusion rates of all membrane-bound proteins including glutamate receptors due to crowding (Santamaria et al., 2010). Cell adhesion complexes have also been identified as diffusion barriers for membrane proteins (O’Brien et al., 1999; Saglietti et al., 2007). Lastly, the actin cytoskeleton is known to mediate receptor positioning (Gudheti et al., 2013) and its depolymerization was shown to reduce glutamate receptor accumulations in spines (Allison et al., 2000). In the following sections, we discuss the role of spine shape on the passive (diffusive) and active (endosomal) transport of receptors.

### The effect of spine shape on lateral diffusion

**Figure 3:**
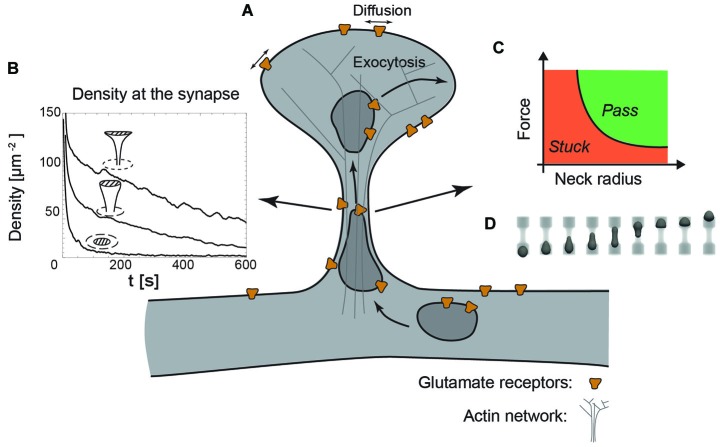
**The dendritic spine as a regulatory system. (A)** Schematic view of a dendritic spine containing recycling endosomes, glutamate receptors and actin cytoskeleton. **(B)** Decreasing the radius of the neck increases the retention of receptors at the synapse, indicated by the time-evolution of the density at the synapse (dashed area) for a planar, stubby and mushroom shaped spine (Kusters et al., 2013). **(C)** Phase diagram indicating that decreasing the neck radius increases the force necessary to transport recycling endosomes through the actin rich constriction. **(D)** Typical sequence of shapes during the translocation of an endosome through the neck, obtained with three-dimensional Lattice-Boltzmann simulations (Kusters et al., 2014b).

Several experiments have shown that two-dimensional diffusion of membrane markers and AMPA-type glutamate receptors is sensitive to the morphology of the dendritic spine (Ashby et al., 2006; Shim et al., 2012). Mushroom shaped spines were found to retain AMPA receptors in the vicinity of the synapse for an increased period of time (Ashby et al., 2006; Ehlers et al., 2007; Opazo and Choquet, 2011, see Figure 3A). These observations have been rationalized by several modeling studies, which showed that the typical mushroom-like morphology of dendritic spines strongly alters the lateral diffusion of AMPA receptors, demonstrating a pronounced suppression of the receptor exit rate out of spines with decreasing neck radius as well as increasing neck length (Holcman and Schuss, 2011; Kusters et al., 2013; Kusters and Storm, 2014). More specifically, the characteristic timescale for retention, the mean escape time of receptors through the neck of a typical mushroom-shaped spines follows a power-law dependence on neck radius *r*,

$$\tau_{\text{escape}}\sim {\left(r_{\text{neck}}\right)}^{-\text{λ}}$$

as well as neck length *l*,

$$\tau_{\text{escape}}\sim {\left(l_{\text{neck}}\right)}^{\eta }$$

where *λ* and *η* are positive constants, whose numerical value depends on the actual shape of the spine (Kusters et al., 2013; Kusters and Storm, 2014).

In combination with an exocytic event in the head of the spine, a decreasing neck radius and increasing neck length effectively increase the confinement of receptors at the synapse, as can be seen in Figure 3B showing the time-evolution of receptor concentration after the release of 1000 receptors at the top of the spine (Kusters et al., 2013). Mushroom shaped spines with the smallest neck radii are thus significantly more effective at retaining receptors. Moreover, the particular shape of the mushroom-shaped spine in combination with receptor trapping at the synapse further enhances their retention. The timescale for an AMPA receptor reaching the synapse may be up to an order of magnitude faster that the time it takes for a receptor to exit through the neck of the spine. Altogether, this modeling study concluded that mushroom shaped spines with an exocytosis site adjacent to the synapse are privileged over others, because they can rapidly and specifically regulate the synaptic AMPA receptor level (Kusters et al., 2013).

Hydrodynamic interactions of proteins with the plasma membrane and the surrounding liquid significantly reduce their mobility. For flat membranes, Saffman and Delbruck (1975) predicted a logarithmic dependence of the diffusion coefficient with the “size” of the membrane, relative to the size of the protein. Recent experimental studies on membrane tubes show that reducing the radius of a membrane tube, which sets the relevant length scale in the Saffman-Delbruck theory, indeed reduced the mobility of both lipids and proteins with a factor of five compared to planar diffusion (Daniels and Turner, 2007; Domanov et al., 2011). The thin and slender neck, typical for mushroom spines, is in that same range of radii as in these experiments by Domanov et al. (2011) and could therefore reduce the mobility of glutamate receptors, compared to that on the dendritic shaft.

The concept of a freely diffusive environment for these receptors, as has been presumed in all the previously described studies, is a very crude approximation of reality. The dendritic membranes on which these receptors reside are, similar to other biological membranes, highly crowded structures (Takamori et al., 2006). Crowding itself is known to significantly decrease the in-plane mobility of proteins (Santamaria et al., 2010). A recent study on the diffusion of steric repulsive particles confined to a cylinder confirmed that, for dense systems, a tubular geometry effectively limits the diffusion of particles along the long axis of the tube (Kusters et al., 2014a). However, how crowding exactly affects the diffusion on highly curved structures remains elusive and will be the focus of future experimental and theoretical studies.

### The effect of spine shape on vesicular transport

Besides the effect of shape on lateral mobility, the overall shape of a dendritic spine also impacts the active transport of recycling endosomes. These endosomes, necessary for the delivery and the retrieval of receptors, have been found to operate both close to the synapse, within the head of the spine and in the dendritic shaft (Park et al., 2006; Wang et al., 2008). Endosome-based delivery of receptors into the head of the dendritic spine does come at a cost: to reach the spine head, they have to cross the actin rich neck, which inevitably causes the endosomes to deform. A recent study that explicitly modeled the translocation of vesicles through narrow constrictions has shown that the force produced by a realistic number of molecular motors is capable of transporting an endosome through constrictions with similar dimensions as spine necks (Kusters et al., 2014b). However, this translocation is highly sensitive to the size of the neck and the applied force. This can be shown in a phase diagram indicating whether an endosome passes through the neck or gets stuck in the constriction; see Figures 3C,D. Although this study did not explicitly model the actin meshwork in the neck, nor the potential deformation of the spine neck itself, it suggests that decreasing the size of the neck, in contrast to its effect on passive diffusion, could hamper the active transport of receptors (Kusters et al., 2014b). Further development of this model requires a careful experimental analysis of the deformations of both vesicles and the spine neck during spine entry events.

## Summary and outlook

In this review we have highlighted existing evidence for a role of spine morphology in the compartmentalization of different important processes, such as receptor trafficking and multiple signaling events. Despite the importance for spine functioning, the exact mechanisms that govern compartmentalization are poorly understood. For example, the extent in which protein diffusion is governed by spine shape alone has remained unclear, because most experiments have so far been unable to directly correlate dynamic readouts with exact spine shape. Importantly, two pioneering studies have recently exploited developments in high-resolution light microscopy to more directly map spine morphology in live experiments and examine its effect on diffusion of free molecules (Takasaki and Sabatini, 2014; Tønnesen et al., 2014). Combined with the mathematical modeling approaches that we described (Kusters et al., 2013), this should allow to dissect the interplay between purely shape-based compartmentalization mechanisms and additional cell-biological mechanisms that confine both signaling and receptor localizations. A better understanding of spine compartmentalization and its implication in plasticity will lead to a deepened and refined model on how synaptic strength is regulated on a molecular level.

## Conflict of interest statement

The authors declare that the research was conducted in the absence of any commercial or financial relationships that could be construed as a potential conflict of interest.
